# myBlackBox: Blackbox Mobile Cloud Systems for Personalized Unusual Event Detection

**DOI:** 10.3390/s16050753

**Published:** 2016-05-23

**Authors:** Junho Ahn, Richard Han

**Affiliations:** 1 National Security Research Institute, Yuseong, Daejeon 305-600, Korea; 2 Department of Computer Science, University of Colorado, Boulder, CO 80309, USA; richard.han@colorado.edu

**Keywords:** unusual event, mobile user, blackbox, behavior pattern, fusion

## Abstract

We demonstrate the feasibility of constructing a novel and practical real-world mobile cloud system, called myBlackBox, that efficiently fuses multimodal smartphone sensor data to identify and log unusual personal events in mobile users’ daily lives. The system incorporates a hybrid architectural design that combines unsupervised classification of audio, accelerometer and location data with supervised joint fusion classification to achieve high accuracy, customization, convenience and scalability. We show the feasibility of myBlackBox by implementing and evaluating this end-to-end system that combines Android smartphones with cloud servers, deployed for 15 users over a one-month period.

## 1. Introduction

Traditional blackboxes [1,2] are used in emergency situations to record the events leading up to a disaster, such as a plane crash, in order to aid investigators in determining the causative factors. They are typically designed to record the most recent data and are ruggedly built to withstand loss of power and extreme physical stress. In myBlackBox, our idea is to adopt the basic blackbox spirit of recording unusual events and to extend this functionality into the mobile phone domain. myBlackBox provides a basic system that efficiently and accurately detects and records “unusual events” of smartphone users, both on the mobile device and in the cloud, based on mobile sensor data.

There already exist some types of human event detection systems [3,4,5,6,7,8] similar to ours, which are used to identify unusual human behaviors and patterns, using activity, video or audio sensors. These systems, however, are either not developed for the mobile phone at all (e.g., they use only a stationary video recorder) or are minimally implemented on the smartphone (e.g., they only use the activity sensor of the mobile device) [3,8]. For our current research, we have designed our system to utilize all of the following sensors of the mobile phone, audio, location, accelerometer and mobile status, simultaneously, in order to detect and log a mobile user’s daily events and then to classify them as either normal or unusual events.

We define “unusual/abnormal events” to be infrequently-generated behaviors of mobile phone users, such as extremely increased or decreased physical activity, infrequently visited locations or unusual audio identification. To decide on the threshold frequency for determining whether an event was unusual or not, we needed feedback from the users. The threshold frequency may vary across users, so to verify our results, we queried each participant in our studies on the mobile device to verify whether a particular user would label an infrequently-occurring event as an unusual event in their life or not. From this survey, we were able to determine, for example, that the majority of the 15 subjects in our research experiment confirmed that the places they visited less than 2% during a one-month time period were places visited that constituted “unusual events” in their normal schedules.

In our previous research, we designed an unusual event detection algorithm [9] using mobile sensor data collected from 20 users over a period of one week. We built and tested this algorithm offline, fusing location-based audio (low level sounds, talking voice, music and loud emotional sounds) and location-based activity (stationary, walking and running) classifiers. The accuracy of the algorithm was evaluated offline; thus, this approach was not deployed nor evaluated in a practical real-world mobile cloud system. However, given the promising initial results of the prior work, we decided to build and deploy a working mobile cloud prototype with actual users, in addition to incorporating algorithmic modifications to account for real-world challenges.

This research paper focuses on the results of deploying the myBlackBox system to real-world mobile users, where we have concentrated on investigating the scalability and efficiency of our system. The myBlackBox system is designed to operate automatically in the background on the smartphone, without conflicting with other mobile sensor data being collected. Furthermore, during our design and testing of our myBlackBox system for real-world application, we used lessons from the deployment to improve upon our fusion algorithm during the testing and deployment phase. We also extended the period of data collection and analysis to a one-month period, in order to better evaluate and verify the accuracy of our algorithm’s operation on the mobile phone as a practical, real-world system.

By building myBlackBox, our goal is to demonstrate the feasibility of extending the blackbox concept to today’s mobile smartphones. We will show that myBlackBox is robust and accurate enough for general purpose applications that need to detect unusual events in a user’s daily behavior. Today’s smartphones possess a wide array of heterogeneous sensors, e.g., location, audio, accelerometer, *etc*. Therefore, our challenge is to devise a practical strategy that fuses together data from all of these mobile sensors in order to accurately detect and record unusual human behavior events of the users of our system. It must also account for other types of limitations imposed by the mobile scenario, such as sporadic disconnection and resource constraints. Fundamental and common to all of these types of applications is the underlying core system of “mobile + cloud” that efficiently detects and logs normal and unusual mobile user events.

This work makes the following contributions to research in this area:

1. Practically-improved classifiers combining location history with audio sensor data, as well as with accelerometer sensor data were developed to identify unusual user events by exploiting personal historical data and filtering noise.
2. A hybrid architecture was practically designed that combines unsupervised classification of location-based audio and location-based activity with supervised joint fusion classification to achieve acceptable accuracy, customization, convenience and scalability.
3. This work identified the best supervised learning algorithm among four popular fusion algorithms for fusing together multi-modal sensor data for identifying unusual user events and characterized its improvement in accuracy over location-based audio and activity classifiers.
4. A complete end-to-end mobile cloud system, called myBlackBox, for efficient detection and logging for unusual user events was implemented and evaluated in a real-world deployment on Android smartphones and a cloud server.

## 2. Related Work

Related work in human event detection systems is concerned with the identification of human behavior and patterns using activity, video or audio sensors. In one research study [3,8,10], the researchers used the activity sensor, which can identify activity and movement patterns [11,12] on the mobile phone, for detecting any unusual falling events of the subjects. This research focuses on detecting falling behaviors of elderly people or hospital patients, but is limited in its application for measuring other activity and behavior patterns of the general population. Other research [4,13] made use of a video camera to record and measure subjects’ behaviors, by installing a video camera in one room where the research took place. However, this approach is very limited to a specific range and fixed location, whereas our mobile application is transportable and easily deployable for multiple users over a wide area and range. In another study, we found audio detection systems being used to detect unusual events in subjects’ immediate environments. The audio system [5,6,7] for detecting unusual, and possibly dangerous, events was a more simplified system than ours, employing audio sensors in a specific area to capture any generated threatening or unusual human voice sounds, such as screaming, shouting, *etc*. The system could detect and classify unusual sound events of people in a prescribed, limited area, but it was only tested in a laboratory setting.

Some GPS tracking systems [3,14,15] have been used to identify unusual locations encountered by Alzheimer patients, who use mobile phones installed with these systems. These systems learn the patients’ daily-visited areas and movement range within their houses or within a prescribed indoor/outdoor area within a certain range and then divide this range into safe and unsafe areas. The patients usually stay within one or two main area ranges, and these systems simply identify when the patients wander into new/unusual, possibly unsafe, locations. This GPS tracking approach is sufficient for purposes of monitoring Alzheimer patients, but is limited for use in applying it to normal mobile users, because it focuses on tracking users’ movement within a very limited range. Normal everyday mobile users visit many more places and move within much wider ranges throughout their daily lives.

Existing unusual event detection applications [16,17] provide family members’ current locations or historical locations to detect unusual location events. Family Locator-Kids Tracker [17] and Family Family Locator-GPS Tracker [16] applications are able to share users’ locations among family members using the GPS on the mobile phone. However, they require family members who use this application to monitor another member and to manually check that member’s location frequently. These mobile applications also only use one dimension of the mobile device’s sensor data.

For our research, we classified audio data using existing audio classification algorithms that can measure combined audio patterns (*i.e.*, low level noise, talking voice, music and loud emotional sounds). Popular algorithms used in these applications to classify audio data are the MFCC (Mel-Frequency Cepstral Coefficients) [18] and GMM (Gaussian Mixture Models) [19] algorithms.

Machine learning techniques are used in a variety of research domains [20,21,22,23] to identify or improve their detection accuracy performance. A misbehavior detection system [20] identifies users who misbehave (*i.e.*, users engaging in pornographic chatting) by using machine learning algorithms with mobile sensors on the mobile video chat application. Another type, a mental health tracking system [21], detects users’ mental health status through the use of camera facial recognition, user interaction logging and social media content information. An emotion detection system [22] can recognize human emotions using sensors that detect facial expressions, electrodermal activity, heart rate and skin temperature. An aggressive detection system [23] classifies human aggressive behaviors using machine learning techniques with audio and video sensors that improve the system’s classification performance by efficiently combining multi-modal sensor data. Each of these system’s domains analyzed varying combinations of multiple sensor data, depending on their research goals, in order to boost their accuracy performance with optimized machine learning algorithms. In our domain, we also used a specific combination of multi-sensor data (*i.e.*, accelerometer, audio, Wi-Fi, GPS and phone status) to develop a practical system that identifies user events with machine learning algorithms in a real-world setting.

A key theme of this research is fusing together multi-modal mobile sensor data to arrive at an improved classification of unusual user events. We investigated tree-based binary fusion classification algorithms that could be used to identify when mobile users encounter an unusual situation or are involved in an unusual event (different from their daily patterns). We compared four popular fusion classification algorithms, Bagging [24], AdaBoost [25], SVM (Support Vector Machine) [26] and CI (Confidence Interval), to find the best fusion algorithm of the four classifications for use in our system. Bagging is a bootstrap aggregating algorithm that uses a machine learning ensemble method to build an improved classification model, using average predictions or majority voting from multiple models. The AdaBoost algorithm is another algorithm that uses the ensemble method to build a strong classification model with weak classifiers to improve the performance accuracy. SVM is a supervised learning algorithm, which is used to build an optimal linear classification model. CI is an algorithmic method, used to determine the optimal interval range in which the probability of a given hypothesis can be said to be true or not. We built a classification model according to each of these tree-based binary fusion algorithms and compared them to find the best fusion algorithm of the four classifications to use in our system.

## 3. System Challenges and Design

### 3.1. Assumptions and Goals

The foremost design requirement for our proposed system is to achieve blackbox-like functionality for mobile users by both detecting unusual user events based on smartphone sensor data and logging those events for later retrieval in the cloud and on the mobile device. In case the mobile device is lost, then logging of data to the cloud acts as a reliability mechanism that can help locate the last known whereabouts of a missing mobile user and/or to recover the history of the mobile device. Additional benefits accrue from having a cloud server store a log of a user’s event trace. For example, parents wanting to monitor the safety of their children can login to the myBlackBox cloud server to inspect the record of activities forwarded from their kid’s myBlackBox mobile application, even if their kid’s mobile device is disconnected/off. Moreover, the parents can create callbacks on the server that notify them when a specific set of circumstances occurs.

Caching logged data on the mobile device brings its own benefits. It helps bridge temporary disconnection issues faced by wireless users even in seemingly well-connected cities. Moreover, if a user is lost hiking in a remote location without their cell phone, the discovery of their mobile device and its logged data can help locate the last known whereabouts of that user, as well as reconstruct a history of the lost user’s actions leading up to that event, which may help in search and rescue.

Our system is designed to satisfy a variety of other design goals. Due to the diversity and dynamics of indoor and outdoor environments, user locations and user activities, we need to accurately identify the unusual events that users generate (with fine granularity and robustness), with low latency and without incurring processing overhead on the mobile device. Further, our system should use a combination of sensors to improve the classification of whether a user is experiencing an unusual or abnormal situation. Furthermore, our system should log seamlessly and automatically in the background and not require much, or any, user intervention. In addition, our mobile application is designed to be sufficiently energy-conserving, so that the phone may operate for at least twelve hours a day with the application running. Finally, we intend to leverage a cloud server for the logging of multiple users’ unusual events in a scalable manner.

Our solution does not make unrealistic assumptions about the existence of specialized infrastructure to assist with any of the above tasks, such as the existence of elaborate sensors. We assume only the capabilities and existence of sensors common to most standard smartphones (e.g., today’s iPhones and Android phones), such as audio sensors capable of capturing sounds, GPS capable of measuring locations and accelerometers capable of measuring activities. We assume that the Wi-Fi works indoors, which we have verified to be practically true in typical indoor settings. We assume there may be occasional wireless disconnection. We do not assume the existence of gyroscopes on the phone, since not all smartphones support them. We expect users to behave in a normal manner, namely carrying and using the phone as they typically would with our application running in the background and exhibiting other usual behavior, such as recharging the phone every evening/night [27].

Our task is then to show how under these limiting assumptions, we can still construct a system that successfully supports our goals of achieving unusual event detection in practical, real-life settings.

### 3.2. Algorithm Challenges and Design

Given these system goals, we are then faced with the following algorithmic challenges.

- How can we classify personal user behaviors using multi-modal mobile data?

**Figure 1:**
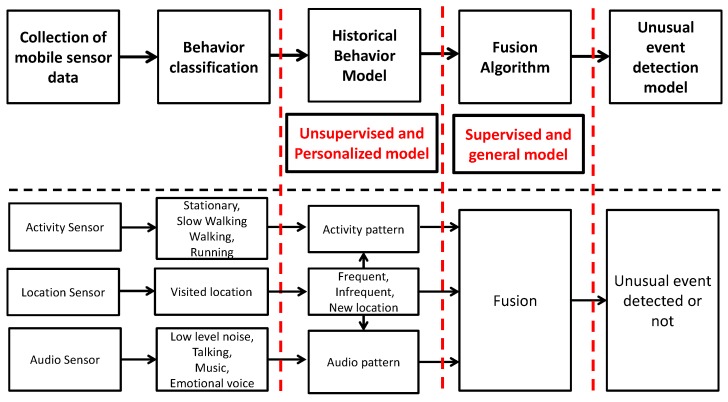
Process for building an unusual event detection model using mobile sensor data.

We confined ourselves to three common mobile sensors, namely location, activity and audio. Figure 1 shows that our approach was to first build single-mode behavior classifiers and to then fuse their results into an integrated decision to identify unusual events [9]. The intent was to exploit the increased accuracy that should result from utilizing multiple sensors to make a collective decision identifying an event. The individual user behavior classifiers categorized mobile users’ activity (e.g., stationary, slow walking, walking, running), audio (e.g., low level sound, music, talking, loud emotional voice) and locations (ranked list of frequently-visited locations).

Based on data obtained from the previous 20 mobile users, we observed that an activity’s normality/abnormality is context sensitive to both location and user. That is, a given user tends to exhibit repeatable activity on a set of frequented locations, such as sitting at work or running in the gym. Thus, simply identifying whether a user is running or not is insufficient for accurately identifying abnormality, which is a function of the user’s location. Instead, it is important to develop a location-based model that identifies what is a user’s normal behavior at each location.

In addition, it is important that a different location-based model be developed for each distinct user. It may be normal for the audio data to reveal loud noises and screaming at a user’s home if that user particularly enjoys watching action/horror movies at home. For another user, this may be highly unusual.

Therefore, on top of the basic single-mode classifiers, we built location-aware audio models and location-aware activity models for each user. To use this approach, we observed that mixed percentage patterns of the classifications follow the normal distribution. We used the Gaussian (normal) distribution to find the probability of how close each subject’s type was to the normal classification probability. The normal Gaussian distribution is bell-shaped, and its probability is calculated by the average and standard deviation. We used this normal distribution to analyze our historical location-based audio data (*i.e.*, audio sets collected in the same location). These are then used to generate early stage classification results based only on pairwise data. To combine all three modalities of data and improve accuracy, namely to combine the results of the location-based audio models with the location-based activity models, we employed fusion algorithms as described below.

- How can we automatically detect unusual user events and thereby minimize inconvenience to the end user, *i.e.*, not require the mobile user to manually label significant unusual events to train the system before it can be made useful to the individual?

As noted above, developing classifiers that are personalized to each user’s normal/abnormal behavior should yield the best results for unusual event classification. However, we wish to minimize and ideally eliminate manual labeling of data by the mobile end user, yet achieve personalized classification. Our approach was to employ a hybrid fusion approach in which unsupervised learning [28] (without labeled training data, *i.e.*, feedback from a user) is employed for the initial phase of location-based audio and location-based activity classification, while a supervised learning (with labeled training data) [29] approach is used for the fusion classifier. Unsupervised learning means we can forgo user training and labeling, yet develop reasonably accurate models of unusual events that are customized to both location and user. These models can be developed on a user’s mobile device in an automated manner, without requiring user input. We then rely on supervised learning at the next fusion stage to boost the accuracy.

Ultimately, does not each mobile user still need to be involved in the training loop to label whether a fusion result is truly a significant unusual personal event? *We avoid this burdensome manual labeling requirement by the following key finding from our research: we can approximate the accuracy of a personally-trained fusion classifier with a classifier trained jointly from a general collection of mobile users.* That is, our research results show that we can develop a general fusion classifier based on supervised learning that is trained by hiring, say, N users/strangers and paying them to label their data and that this general fusion classifier achieves a similar accuracy to a personalized fusion classifier. In this way, we eliminate, as well, the need for the user to train the fusion classifier.

**Figure 2:**
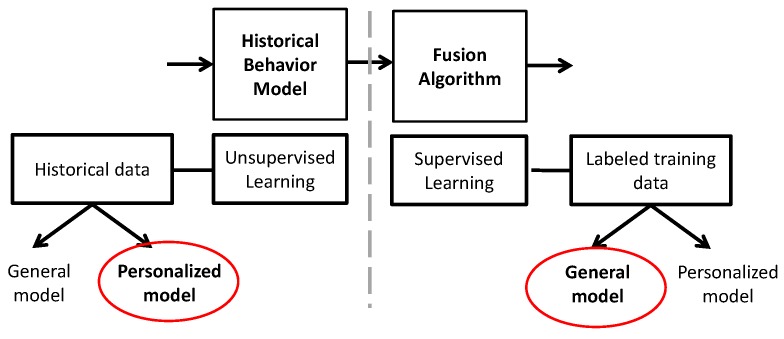
Diagram of unsupervised and supervised learning algorithms, with general *versus* personalized model choices.

Figure 2 zooms in on the two critical classification stages of Figure 1 and summarizes the combined decision-making process of our hybrid fusion design for the myBlackBox system. We now see that the pattern recognition and fusion algorithm steps comprise the unsupervised learning stage and supervised learning stage, respectively. In the pattern recognition stage, which is based on cumulative historical user data, we chose a personalized model over a general model because we needed to measure individual users’ behavior patterns over time as accurately as possible. If we had chosen a general model, it would not have been as sensitive to each individual’s unique daily behavior patterns. For example, for this unsupervised learning algorithm, we measured the probability of each individual’s 30-min segments of personalized behavior pattern data as to whether these fell within their user-specific historical normal range of cumulatively-measured daily behavior patterns. In the fusion stage, which is our supervised learning algorithm that uses labeled training data (user subjects’ feedback on our classification algorithms), we determined that developing a general unusual event model based on generic users’ labeled data achieves basically commensurate accuracy in our system compared to a personalized model.

Therefore, our hybrid fusion design is hybrid in two dimensions; namely, it combines both unsupervised and supervised learning and also combines both personalized and general models of unusual user event behavior. The end result is a hybrid fusion design that is both accurate and convenient for the mobile end users.

### 3.3. General *versus* Personalized Fusion Model

A key advantage of the general model is that if its accuracy can be shown to be roughly equivalent to a personalized model of classification, then we can substitute the general model in place of the personalized model and, thus, avoid requesting that the user manually label periodic data as indicating an “unusual event” or not, in order to develop a personalized classification model under supervised learning. If we can show that the general model functions at a similarly high equivalent accuracy as the personalized model, we can be confident that our hiring of a small set of completely unrelated users to develop the general model is sufficient and, thus, incorporate this general classifier into the mobile application to achieve almost equivalent unusual event detection as is attained by the personally-trained classification model.

**Figure 3:**
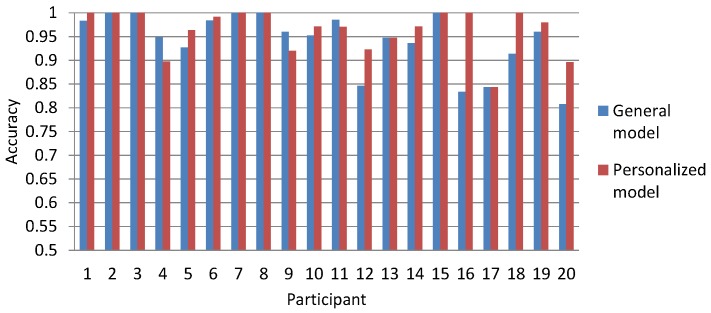
Accuracy evaluation of the general model *versus* the personalized model, for identifying unusual mobile user events.

To analyze the accuracy and performance of the general model compared to the personalized model, we used the highest performing algorithm for the general model analysis, the Bagging algorithm, and the highest accuracy measurement of each individual’s set of data for the personalized model. Figure 3 shows the resulting accuracy measurements achieved for each participant’s set of data, side by side, when the general model was used *versus* when the personalized model was used. To obtain the accuracy measurement for each individual’s data using the personalized model, we chose the highest accuracy measurement among that person’s data. To obtain the accuracy measurement for the general model for each individual user, we used the Bagging algorithm results based on the combined accuracy measurements from the training data of the set of 19 other users, excluding the chosen user’s training data.

Overall, we found the average personalized model-based accuracy of 0.96 to be slightly better than the average of the general model-based accuracy (0.94), by 0.02 points. Although for the personalized model, the accuracy measurements were higher for 50% of the users (10 of them); the accuracy measurements yielded using the general model were higher (15%) or the same (35%) for the remaining users; as shown in Figure 3. Although the accuracy of the general model was a little lower than that of the personalized model, we noted that its accuracy was sufficiently high enough and comparably enough in performance across all users to be confident in adopting it for our research purposes. For these reasons, we adopted the general fusion model for building our myBlackBox system and for making it more scalable and usable for users of our system.

### 3.4. System Design Challenges

In addition to algorithmic challenges, we also faced the following system challenges given the design goals of our system:

- How do we devise an end-to-end system that combines mobile smartphones with remote cloud execution, while balancing the limitations of the resource constraints of mobile devices with the desire for our cloud server to scale to thousands of users?

Our myBlackBox system was initially designed to handle the data of hundreds of mobile users at one time [9]. At first, we were challenged in determining where to place the execution of the myBlackBox algorithms and procedures: on the mobile phone or on the server. The mobile phone has more limited resources than the server, such as lower battery power and a slower CPU; however, the server cannot always handle the processing or network traffic overhead of a large number of users simultaneously, when the algorithmic processing takes inordinate amounts of time for each user’s data. Our mobile application has to collect and send mobile sensor data periodically to either the phone or the server for processing. The myBlackBox system also utilizes classification algorithms (location, activity and audio) and a fusion algorithm, which need to be run on either the phone or server, to detect unusual events occurring in a user’s daily life.

To support blackbox-like remote logging to the cloud server in a scalable and bandwidth-efficient manner, we choose to send only summaries of the latest data from the mobile to the cloud server. We set up the mobile application to classify each individual mobile sensor’s data and to summarize and store the results, using our fusion algorithm, on the smartphone. Each mobile user’s summary results are then sent to the server. In this manner, we found we could reduce the overhead of the server by running our application on the users’ mobile phones. In addition, we tested and emulated this process and found that our system could handle a maximum of 20,000 mobile users on our server.

### 3.5. System Architecture

**Figure 4:**
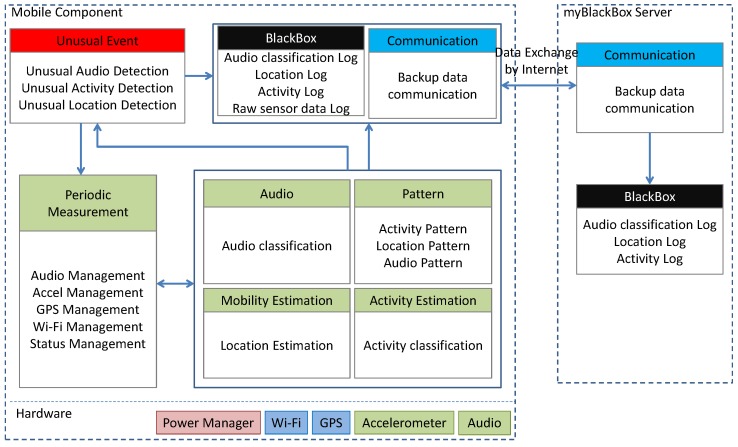
Architectures of the myBlackBox mobile component and the myBlackBox cloud server.

Given these above system goals and challenges, we made the following design decisions. The mobile and cloud server components are assembled according to the architecture as shown in Figure 4. In this architecture, the myBlackBox application fuses together information from sensors, like the microphone, GPS and accelerometer, to estimate the likelihood of an unusual event. The fusion algorithm is built on top of individual classifiers for each of the three following sensor dimensions: audio, location and activity. These three sensors thus far have provided relatively strong indicators of unusual mobile user events, though we continue to explore other sensing dimensions.

The architecture of myBlackBox consists of two logical components:

**Mobile component**: This component runs on the user’s smartphone. The mobile component implements two important functions. First, it implements an unusual event detection system that automatically fuses the phone’s sensors’ measurements in order to detect any unusual events experienced by the user. Second, it implements blackbox functionality to record all sensor measurements that are stored locally as clues and evidence on the phone. This information is then automatically sent also to the myBlackBox server in the cloud.

The myBlackBox application reads sensor data from the mobile phone in order to identify both usual and unusual mobile user events. The mobile application continuously activates the accelerometer sensor to measure a user’s activity and periodically activates the audio sensor every 5 min to record audio data as a wav file for 15-s periods. After collecting the audio data, it analyzes the audio file with the MFCC and GMM algorithms and classifies the audio as numeric data (e.g., (1) quiet sound; (2) talking; (3) music; (4) loud sound). It also records the users’ location with either Wi-Fi, every 3 min, or with the GPS sensor every 5 min, when Wi-Fi is not available. We determined that our mobile application on the smartphone cannot share the audio sensor capabilities simultaneously with other mobile applications (e.g., phone calling, game playing, Skype, *etc.*). Whenever our myBlackBox application is activating the audio sensor, other applications (such as phone calling) cannot utilize the audio sensor. Because of this issue, we decided it would be best not to conflict with any possible audio sensor use of the myBlackBox research participants who were using our system. Thus, we did not activate the audio sensor whenever the mobile user was interacting with their phone in any way: e.g., turning on the screen (e.g., to surf the web, text, *etc.*) or calling. Using the methods described above, our application continually collects 30-min segments of a mobile user’s location, activity and audio sensor data on the mobile phone and then, with the 95% Confidence Interval (CI), determines whether the user is experiencing an unusual or normal daily event.

**Figure 5:**
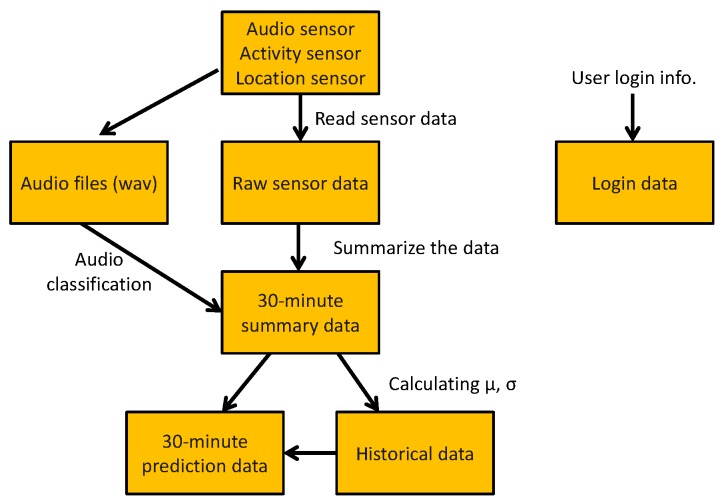
Diagram to store sensor data on the mobile phone.

Our mobile myBlackBox application also provides a blackbox-like functionality to periodically store the sensing data and classification data of a user’s audio, location and activity on the database of the smartphone, as shown in Figure 5. For the audio sensor data collection and storage, the audio is stored as a wav file with a different file name every 5 min. We design five tables (raw sensor data, 30-min summary data, historical data, 30-min prediction data and login data) on a database of the smartphone to store the raw sensor data and our analysis data. We build the tables to store raw sensor data every 5 s, collected from the mobile’s audio, activity and location sensors, whenever there is data readily available from these sensors. Every 30 min, we build a table for the percentage-based summary data, which is calculated from the raw activity, audio and location sensor data. This table stores a log of the percent of time a user spends engaged in various activities and a user’s variation in audio and movement patterns. This 30-min data from the summary table is used to build the historical data table of the mobile user. The historical table includes the averages and standard deviations of a user’s cumulative sensor data calculated from the summary table data, beginning at Day 4 up to a period of one month, based on the user’s normal distribution patterns. We also build a prediction table to store the probability values of the 30-min summary sensor data for each of the audio, activity and location measurements. Using these probability values, we then store a binary classification result of whether the 30-min period of data constituted a normal or unusual event for the user. Lastly, we design a login table, where the mobile user’s password and login ID information are stored. This table is used to provide access for users to our system’s website, where they can view graphs of their historical personal behavior data. The 30-min summary data and 30-min prediction are stored for up to 30 days and then removed from the mobile device’s databases. Raw sensor data is stored on the phone for up to three days only. Users can easily access their raw data and summary and historical data on their own smartphones during these time periods. We do not store the raw sensor data and audio files on the server, because of user privacy issues and some users’ limited mobile data plans.

**myBlackBox Cloud Server**:The remote blackbox server component of our system also records and stores all event data collected from each mobile user, providing an extra degree of redundancy in case the mobile device is lost, destroyed or stolen. Logged data, clues and evidence can be recovered despite disconnection from the mobile device. The server also provides a web service for mobile users who wish to access their own historical personal behavior data through a web browser (or for parents to monitor the safety of their children).

## 4. End-to-End myBlackBox Mobile Cloud System

To study the feasibility of the myBlackBox concept and hybrid fusion classifier design, we built an end-to-end myBlack mobile cloud system that enabled both mobile users to collect and monitor their own daily behavior pattern data on their phones and a cloud server to collect users’ summary behavior data. We implemented the myBlackBox mobile application and published it on the Android market to provide this behavior-tracking application to users and to test real-world feasibility for use on the mobile phone. We also developed a cloud server that stored each user’s summary data in a database and provided users password-protected access to a display of their historical behavior-pattern data upon request. Our mobile application was implemented on the phone to automatically transfer each mobile user’s summary data from their phones to the cloud server every 30 min. Previously, we had tested our fusion algorithm for this system manually on a server, but had not implemented it on a smartphone until now. Before collecting data from users through the Android Market, we had our research approved by our university’s Institutional Review Board (IRB) [30]. After collecting one month of myBlackBox system data from 15 mobile users who participated in our Android Market study, we were able to evaluate the feasibility of our fusion algorithm working on the smartphone. Our myBlackBox system is still published on the Android Market, and data are currently being collected.

**Figure 6:**
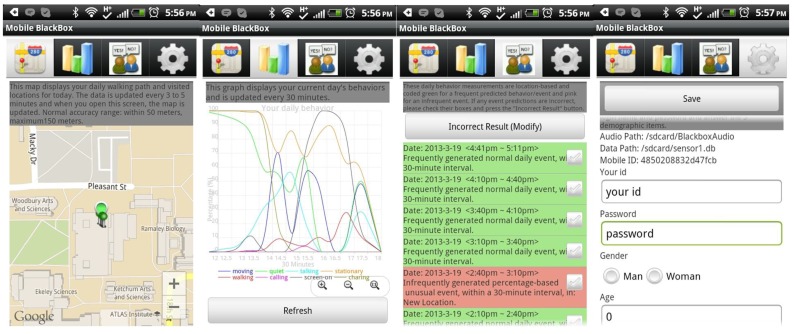
Screen shot of the myBlackBox application.

The myBlackBox mobile application [31] consists of four screens available to the users. Figure 6 shows these four screen shots of the myBlackBox application. The first three screens display the user’s current behavior data. Screen 1 shows the user’s daily map of locations visited during the most recent period. The screen displays up to 100 data points, and the data are updated every 3 to 5 min. Screen 2 shows the user’s behavior patterns for a 24-h period. The daily behaviors collected from each user are based on percentages of occurrences of different user activities logged in a 30-min period. Data were collected from the users’ audio, location, movement activity, *etc.*, measured by the sensors on the phone. This second screen is also scrollable, from the first to the last hour of the current day, and it was refreshed at midnight of each day. The third screen shows summary results of each 30-min segment of users’ behavior data, with our system’s daily event prediction of whether this 30-min period constituted a normal event or an unusual event in the user’s daily schedule. The fourth screen was used initially to collect users’ login information for accessing our web server and summaries of their historical behavior data. We also collected non-mandatory background information on this screen from users who were willing to provide it, on: gender, profession and age. Users only needed to provide a login and password on this screen if they wished to access their historical data on our web server.

**Figure 7:**
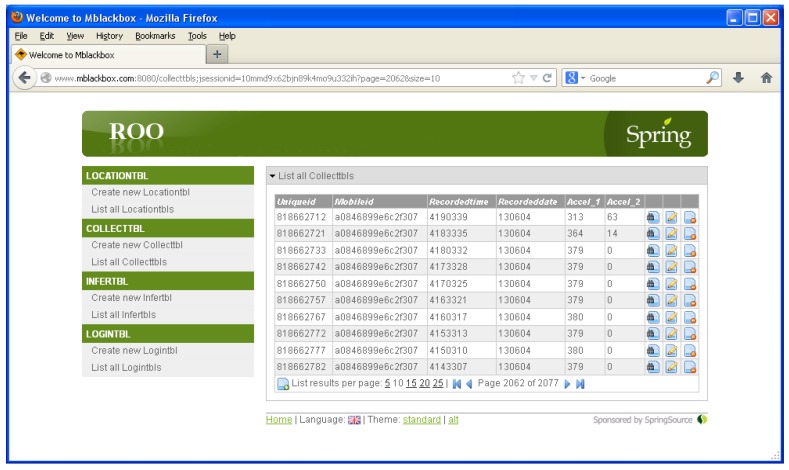
Collection server using Spring Roo.

**Figure 8:**
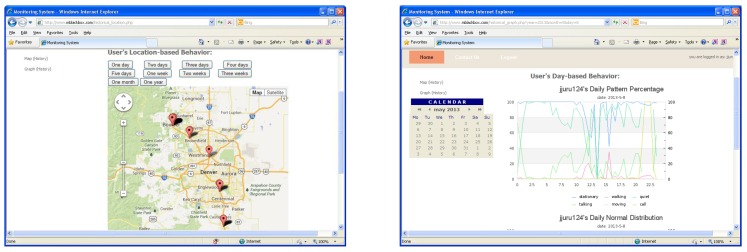
Web server using MongoDB. (**left**) A graphical map; (**right**) A multiple line graph.

For the myBlackBox server, we built two components: a collection and a web component. We installed our system and a database in a cloud server in our laboratory. We have assigned 2 GHz 1 CPU, 2 GB memory and 10 GB HDD for our system server. The collection component of the server retrieves and logs sensor data from users’ mobile phones. It also stores the summary data, processed on the phone, using the sensor and prediction data, which is analyzed every 30 min on each smartphone. The mobile application then compiles these summary data as an XML-based Json file and then sends it to the collection component of the server. The server receives the Json data using the Spring Roo framework [32] and stores it in a MongoDB [33] database, as shown in Figure 7. The web component [34] of the myBlackbox server accesses the MongoDB and processes the data in order to display the users’ location and activity data as a geographical map and line graph, respectively. The mobile users can then view up to a month’s worth of historical record data, summarizing their personal behavior patterns, which include a map of visited locations, activity and audio patterns and mobile phone status, as shown in Figure 8.

For the purposes of this research study, we focused on analyzing one month of data collected from 15 subjects who utilized our Android Market-deployed application continuously for a four-week period. We collected and analyzed both location and activity measurements of the users. The users’ location data were collected using the GPS and Wi-Fi on their phones (e.g., Samsung, HTC), and users’ daily activities and behaviors were collected using the phone’s sensors: accelerometer, orientation, proximity, audio and light sensors. Initially, we tried to run the mobile sensors at all times and to save the data in the mobile phone’s database to capture a continuous record of users’ behaviors in detail [9]. However, the mobile sensors were power hungry, and the storage space is limited on the mobile phone; so, we adjusted the recording time length of use. To conserve battery power, we set our program to measure accelerometer-based activities (e.g., walking, jumping, shaking phone, *etc.*) every five seconds and to obtain and record subjects’ locations using GPS every 5 min and Wi-Fi [35] every 3 min. We also recorded all audio sounds in the users’ immediate area on the mobile phone for 15 s, every 5 min, and stored the orientation, proximity and light sensor data along with these recorded sounds to determine the user’s behavior related to the audio sounds. The battery power of the mobile phone, when running this entire application, lasted for about 13 h. This time was enough to measure a user’s daily behavior patterns and activities, because the users were normally back at home in the evenings and able to recharge the phone at the end of each day.

## 5. myBlackBox Performance Evaluation

In this section, we evaluate the performance of the deployed end-to-end myBlackBox system for the 15 mobile users, whose data were collected over a one-month period.

### 5.1. Accuracy of the Fusion Algorithms

Based on the one months’ worth of data collected from our end-to-end real-world deployment, we investigated the eight behavior patterns of our mobile users (low level background noise, talking voice, music, loud emotional voice, stationary status, slow walking, walking activity and running activity), measured by the audio and activity classifiers, with our four fusion algorithms to determine the best algorithm of the four classifications for identifying whether a mobile user is involved in an unusual or a normal situation. We used a fifteen-day period of training data and a fifteen-day period of testing data to analyze a 30-min segment of the seven days’ pooled data for the 15 mobile user subjects. The 15 subjects’ training data were used to train four classification models designed to detect unusual events. We used four algorithms (Bagging, AdaBoost, CI 95 percent and SVM) for our unusual event classification model development. For each algorithm in our model, we first used the training data (comprised of ground truth data and the fifteen days of the 15 subject’s collected behavior pattern data) to build our four classification models. We used these models as the baseline from which to analyze the testing data to see which of the four algorithms predicted the best overall combined performance for recall, precision and accuracy measurements for analyzing the audio and activity data.

**Figure 9:**
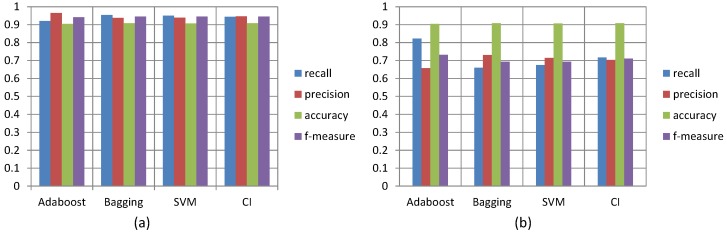
Precision, recall, accuracy and f-measure of four classification algorithms (**a**) for detecting normal events and (**b**) for detecting unusual events.

Figure 9a shows the precision, recall, accuracy and f-measure (combined recall and precision) performance measurements of the four classification algorithms when using them to detect normal events. We found that the best algorithm of the four fusion classifications for detecting normal events was the Bagging algorithm, with the combined performance measurements the highest of any of the other three algorithms: 0.95 recall, 0.93 precision, 0.95 f-measure and 0.91 accuracy measurements. The least proficient performing algorithm of the four was the AdaBoost algorithm, with a 0.91 recall, 0.96 precision, 0.94 f-measure and 0.90 accuracy measurement.

Figure 9b shows the performance measurements of the four classification algorithms when using them to detect abnormal/unusual events. Once again, the Bagging algorithm was found to be the best performing of the four algorithms for detecting unusual events with the highest accuracy measurement of 0.91 and with 0.66 recall, 0.73 precision and 0.69 f-measure measurements. The least proficient performing algorithm of the four was the AdaBoost algorithm, with a 0.82 recall, 0.65 precision, 0.73 f-measure and 0.90 accuracy measurement. We found that the low-complexity CI algorithm, with a 95 percent CI, provided the second highest results for our model and was only slightly less optimal than the Bagging algorithm. In addition, the CI algorithm performed as consistently and efficiently as the Bagging algorithm. Because its performance was highly comparable to that of Bagging and because of its lower complexity of the four fusion algorithms, we determined it to be the most suitable hybrid fusion algorithm for the implementation of our myBlackBox application on the mobile phone.

### 5.2. Noise Removal

We based our CI-based hybrid fusion algorithm computations for each individual user of our application on the user’s normal distribution of behavior pattern data, collected for the duration of a one-month period in the same frequented locations. In our previous algorithmic research, we found that the confidence interval of 95 percent yielded the second best results when comparing the accuracy of various algorithms: Bagging, AdaBoost, SVM and confidence interval. We chose to use the CI fusion algorithm, with a 95 percent cutoff, for our fusion algorithm feasibility study of the real-world application on the smartphone.

When we analyzed the 15-participant data, for 30-min segments that were based on the activities of users who were stationary during these segments, we identified two activities, user game-playing and phone calling activities, which yielded false positives of unusual event detection for our 95% CI algorithm. When we initially included these two stationary user events, we found unexpected “noise data” (false positive identification of unusual user events) in our results that decreased the accuracy of our normal distribution calculation for each user, because these activities were incorrectly identified as unusual events when they were actually normal events. For example, the same measurement results are achieved whenever user-movement activity data indicates that a user is stationary (sitting, with phone stored in the pocket), as when a user is stationary and using their device to play a game on their smartphone. With stationary user-game playing activity, incorrect unusual event detection is indicated compared to what normal activity would be expected when the user is just sitting stationary with the phone on their person. If we were to include both instances of stationary user activity (sitting and game-playing), the accuracy of the normal distribution calculation would be significantly decreased. This example illustrates the difficulty in measuring any user-phone interaction activities accurately, such as texting, web-surfing, skyping, *etc*. In addition to our identification of the “noise” data generated with user-phone interaction activities, such as game-playing, texting, *etc.*, we discovered that the user phone calling activity also could not be incorporated into our normal distribution measurement accurately. We found that the myBlackBox application on the mobile phone cannot record audio simultaneously while a user is making a phone call, because only one application can access the audio device at the same time. Additionally, when users charge their smartphone, there is no activity generated, and if we were to include this charging activity data in our normal distribution, these would interfere with normal and unusual event detection.

Given our above-mentioned findings, we determined that each of these activities, user-mobile phone interaction, phone calling and battery charging, could all safely be classified as normal user events, but that they contributed extra noise to our historical Normal Distribution (ND) calculation, so we eliminated all of them from our measurement. To remove these incorrectly-classified and difficult (or impossible) to measure user events, we used status sensors on the phone to check whether the mobile user is calling, charging or turning on the screen. When the mobile phone is charging, we did not include this activity in our historical ND measurement, but we did access the audio sensor and collect any audio data that were generated. For example, if a user charged his or her phone at night by his or her bed, we could collect user sounds generated, such as a low regular breathing sound *versus* talking voice sounds (e.g., in their sleep or talking near the phone). Similarly the audio sensor collected user voice data, while the phone was charging on a counter or table, whenever the user or another person was standing next to the phone. With a combination of the status sensor, determining that a user had turned on his or her screen (e.g., to play a game, web-surf, text, *etc.*) or initiated a voice call, and the audio sensor detecting the user’s voice, we detected periods of user mobile use or calling and eliminated the audio and these activity patterns from our normal distribution’s calculation of unusual event detection.

**Figure 10:**
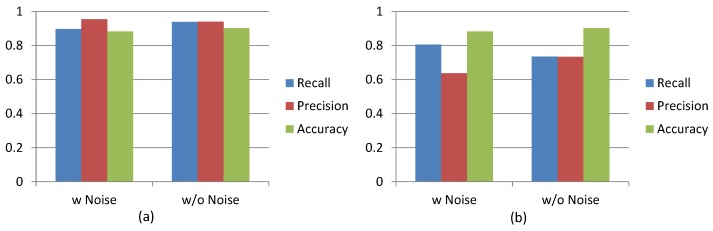
Average of precision, recall and accuracy for detecting (**a**) unusual events and (**b**) normal events using CI.

We investigated three accuracy measurements, accuracy, precision and recall, for detecting unusual events of mobile users’ daily behavior patterns, when the 15 participants of our study carried their mobile phone for a period of one month. We analyzed and compared the measurements in two instances: (1) using the original collected data; and (2) after identifying and removing “noise” data (e.g., phone calling, game playing, charging). Figure 10a shows the averaged measurements, across the 15 mobile user participants, for the precision, recall and accuracy probabilities of correctly detecting normal events, using the CI. By removing noise data for this scenario also, we also improved the overall accuracy and recall measurements by reducing the percentage of false-negatives for predicting normal events. Figure 10b also shows that our method of removing noise data from unusual event detection measurements was successful. When we analyzed the original raw data of the 15 mobile users, we found the average accuracy measurement to be 0.88, but after removing the noise data, the accuracy increased to 0.90. This method, of removing the noise data, also improved the average precision measurement by decreasing false-positives that indicated unusual events, when their were none.

### 5.3. myBlackBox System Evaluation

**Figure 11:**
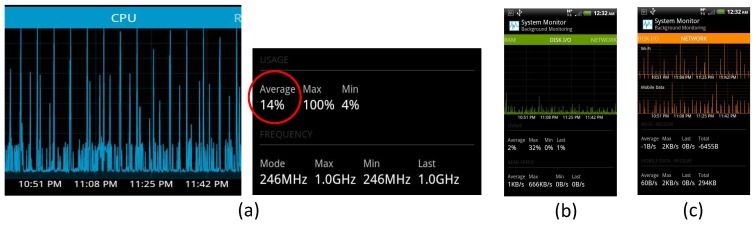
Evaluation of (**a**) CPU, (**b**) network and (**c**) storage I/O usage of the myBlackBox application.

We analyzed the CPU usage of our mobile application using the System Monitor mobile application [36] that can log the CPU usage of mobile phones. Figure 11a shows the CPU usage of the myBlackBox application that we monitored for an 80-min period. We operated our myBlackBox application, while running the System Monitor application as a background service. For about 90 min, our application collected and measured the summary data and prediction data that were collected over three 30-min periods. The average CPU usage was only 14%, which should minimally interfere with most other mobile applications. However, when classifying the recorded audio files (every 5 min for 15 s), we noted that the CPU usage increased to 100% for 7 s. We also investigated the data transfer usage between the network and mobile phone’s storage I/O when operating the myBlackBox application for approximately 90 min, as shown in Figure 11b,c. The average usage of storage I/O of our application, operating on the mobile device, was 2%, with a maximum usage of 32%, a maximum of 666 kilo-bytes per second and an average of 1 kilo-byte per second. We also measured the total amount of network usage and found that the combined usage of both our myBlackBox application and the Android operating system was 294 kilo-bytes during this 90-min monitoring period. In this experiment, the myBlackBox mobile application continually and consistently used 14% (87 MB) of the smartphone’s memory.

We also emulated the performance of our myBlackBox application and server to investigate the scalability issue. The myBlackBox mobile application classifies user events using mobile sensor data collected every 30 min. The application generates the summary data and the prediction data, which are then sent to the server; the size of this data file is 1.5 kilo-bytes. We implemented a mobile application, and it emulated sending the data from mobile phones to the server for a period of 30 min, with an increasingly larger and larger number of potential mobile users of our system, *i.e.*, 100 users, 200 users, ..., up to 1000 users, and then predicted the result up to 20,000 users. Each group of 100 users’ sets of data that were sent to the server took 9 s, and the data transfer time from the phones to the server for each of the other increasingly larger groups was consistently increased at the same ratio, when emulated. The server successfully received all of the data from the increasingly larger groups of users. Based on this analysis, we found that our server can handle a maximum of 20,000 mobile users’ data sent to it during a 30-min period, at which point, it needs to be ready to receive a new set of data for the next 30-min period of mobile user data collection.

**Figure 12:**
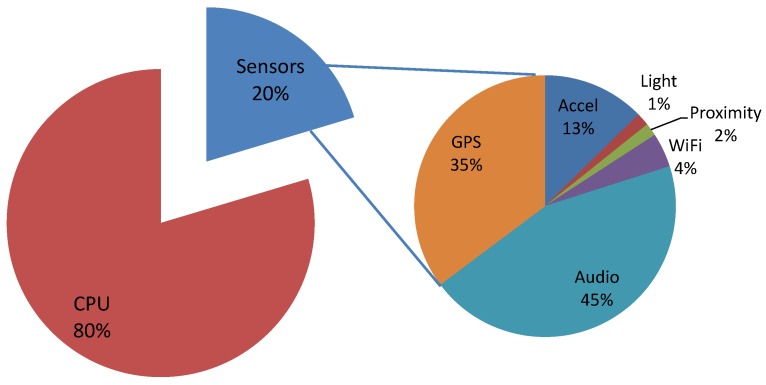
myBlackBox application’s battery usage for one 30-min period.

To study the energy usage of the mobile myBlackBox application, we measured battery lifetime and found that the battery consistently lasted for about 13 h when running the entire application. To analyze how much battery power is used by the CPU and sensors of the mobile phone when running our application, we measured the battery usage of our mobile application using the Power Tutor [37] application and Power Profiles for Android [38], which can log the battery usage of mobile phones, provide battery usage statistics and show sensors’ power usage on the smartphone. Figure 12 shows both the overall CPU *versus* sensor mobile battery usage and the individual sensor battery usage (*i.e.*, GPS, Wi-Fi, audio, accelerometer, proximity, light) on the smartphone. Twenty percent of the battery usage was related to the mobile sensors, and the remaining 80% of the battery usage was utilized by the CPU. The 80% CPU battery power usage is necessarily high because our application works continually as a background service at all times to collect, measure and classify the various types of sensor data. In the future, we plan to further enhance our application’s design to conserve more CPU usage, so that the mobile device’s battery will last longer than 13 h.

## 6. Discussion and Future Work

Future expansion of our system could be achieved by more widely deploying it on the Android Market, while also adding another functionality to the mobile application that queries the users to provide additional information as to which of the unusual events detected constituted actual emergency situations (e.g., health, fire, crime situations). This further research could help us to expand our system’s application for use in accurately identifying real-life emergency situations occurring in a mobile user’s life. We would be able to analyze the data, collected from this large body of users, to study the correlation measurements between the unusual events detected and the verified emergency situations. From these data, we would hope to be able to further refine our system to someday operate safely and accurately in the real world as an emergency-identification mobile application. Once its real-world accuracy in identifying emergency situations has been fully tested and evaluated, a further enhancement would be to add the functionality of automatically alerting emergency personnel in cases of recorded emergency situations. Our hope is that, with further research and refinement of our system, we can provide a myBlackBox system for wide use among mobile users that can help protect and rescue them when encountering emergency situations, such as crimes or accidents.

Our current mobile application’s battery power requirements are limited to a duration of 13 h of operation time, before needing to be recharged. Currently, our application only records and collects sensor data on a periodic schedule. A future design improvement for our system is to build an adaptive algorithm that can collect the most comprehensive amount of mobile sensor data at a time, while conserving the most amount of battery power possible. Our research must take into account the challenges of this tradeoff and, thus, design an adaptive algorithm that can automatically adjust the mobile battery power usage according to adjustable periodic sensor data readings that are dependent on actual user event-type detections.

Our system is currently designed so that all mobile user event data are collected and summarized on the mobile phone, then sent to the server as summary data files. Increasing the recording time on the mobile with our current system is not feasible given the battery limitations of the phone. An additional future design implementation of our system might be to add another functionality to the mobile application that sends all raw sensor data of unusual events to the server, recording this event for its duration up to a subsequent normal event detection. Adding this enhancement would increase the blackbox functionality of our system to collect more historical details of any unusual events recorded. This functionality would provide wider access to clues and evidence regarding possible emergency situations.

Our current myBlackBox system is location-based and, thus, is limited to only being able to detect and identify events as unusual *versus* normal mobile user events whenever a user is in a stationary position. Our system cannot currently detect or classify unusual user events when a user is transitory, moving from one location to another. Initially we tried to incorporate this capability in our system, but found that the data were irregular; and ground truth data collected from some initial participants were also irregular in how they identified normal *versus* unusual walking paths. For future work, we hope to find a way to expand our system to also operate accurately for users when they are in transit, moving from one location to another.

Our myBlackBox system is currently limited to location-based prediction of events as unusual or normal events for mobile users of the system. A future enhancement would be to add time-based historical prediction to the location-based functionality of our unusual event detection system. The system currently collects historical location-based data to predict the future user events using all of the classification data collected in the same location. If we also divide the historical data into several time-based historical datasets in the same location, we would improve the prediction accuracy. For future work, we hope to find a way to expand our system to also operate accurately for users when they visit the same location over different time periods.

Although the myBlackBox system currently identifies unusual events of most mobile users with high detection accuracy, it does not always accurately classify unusual events for all of the mobile users. For further scalability of our system on the mobile market, we would need to address this issue and, at the least, provide some type of identification of the users for whom the unusual event detection accuracy was lower than optimally desired. Otherwise, mobile users with low accuracy prediction of unusual events might be dissatisfied with our system and opt out of using it and/or rate it poorly on the Android Market. To increase the usability and scalability for our system, we hope to develop a way to identify the mobile users whose unusual event detection had low accuracy and to automatically notify them of the results. We would also query them through the mobile interface to ask for feedback on the incorrectly-identified unusual events in order to also further improve the overall accuracy for all users of our system.

Additionally, during the algorithm evaluation phase of our research, the AdaBoost algorithm was found to be better performing than the others for specifically detecting unusual events within the one-month period of mobile users’ data, because of its higher recall measurement. This finding has implications for further research and potential inclusion of AdaBoost for enhancements to a system such as ours.

Some additional system enhancements may benefit future deployments of myBlackBox. More efficient memory usage could be explored. Furthermore, peak CPU usage could be lowered by devising an algorithm that would spread classification over a greater time period. This would mitigate interference even during peak usage with other mobile applications.

## 7. Conclusions

We have demonstrated that the practical and scalable myBlackBox cloud system can be used by mobile users on their own smartphones to identify unusual and normal event occurrences in their daily life environments. Our classification model was also the result of testing various binary fusion classification algorithms (Bagging, AdaBoost, SVM and CI) to find the most efficiently-performing one for our system’s application in the real world. Although the CI-based hybrid fusion algorithm was found to be the second best performing of the four algorithms evaluated, we determined it was sufficiently accurate for use with our application and that for purposes of our myBlackBox system, its computational complexity was simplest to implement on the mobile device. Future potential expansion and enhancement for our system might include adding the functionality of automatically alerting emergency personnel in cases of recorded emergency situations on individual users’ smartphones. Our hope is that with further research and refinement of our system, we can provide a myBlackBox system for wide use among mobile users that can help protect and rescue users of our system in emergency situations, such as situations involving crimes and accidents.
